# Prof. Jeffries Wyman, Boston

**Published:** 1874-09

**Authors:** 


### Prof. Jeffries Wyman.

Prof. Wyman died at Bethlehem, N. H., Sept., 4th, from consumption. His decease was sudden, and was induced by an excessive hemorrhage from the lungs. Prof. Wyman was the son of Dr. Rufus Wyman, and the brother of Dr. Morrill Wyman. He was born Aug., 11th, 1814, and graduated at Harvard, in 1833, graduating in Medicine at the same institution. After receiving his degree of M. D., he went to Europe, and spent two years in Paris, pursuing his studies. In 1843 he was called to Richmond, Va., as Professor of Anatomy, but returned to Harvard in 1847 to accept the chair of Anatomy in that institution, a position which he has filled with honor ever since.

Prof. Wyman was in every sense a scholar, and his writings were received as authoritative by scientists the world over. His writings have been mostly devoted to Anatomical subjects, at least in this department he has gained his widest reputation. In 1849 he delivered twelve lectures upon comparative anatomy, which were published. He also has published papers upon spontaneous generation, on the asymmetry of the human cranium, on embryology, and the anatomy of the nervous system. It will be also remembered that Prof. Wyman was the first to described the skeleton of the Gorilla.

Prof. Wyman has for many years been a sufferer from consumption, but it was hardly suspected that he would follow so closely the footsteps of his intimate friend Agassiz.

